# Supplementary material for: Ex vivo expansion potential of murine hematopoietic stem cells is a rare property only partially predicted by phenotype
Source: eLife. 2024 Mar 6;12:RP91826. doi: 10.7554/eLife.91826 (PMC10942641; doi:10.7554/eLife.91826)
Supplement: Supplementary file 1. [file elife-91826-supp1.docx]

**SUPPLEMENTARY FILE 1**

***EX VIVO* EXPANSION POTENTIAL OF MURINE HEMATOPOIETIC STEM CELLS: A RARE PROPERTY ONLY PARTIALLY PREDICTED BY PHENOTYPE**

Qinyu Zhang^1^, Rasmus Olofzon^1^, Anna Konturek-Ciesla^1^, Ouyang Yuan^1^, David Bryder^1^

^1^Division of Molecular Hematology, Department of Laboratory Medicine, Lund Stem Cell Center, Faculty of Medical, Lund University, 221 84 Lund, Sweden.

Corresponding author:

David Bryder, DMH, BMC B12, Lund University, 221 84 Lund, Sweden +46706423951, [David.Bryder@med.lu.se](mailto:David.Bryder@med.lu.se)

Qinyu Zhang, DMH, BMC B12, Lund University, 221 84 Lund, Sweden, +46462220313, Qinyu.Zhang@med.lu.se

**~~SUPPLEMENTAL TABLES~~**

**Supplementary File 1a. Contents of murine HSC media for *in vitro* culture**

|  | **Final concentration** | **Provider** |
| --- | --- | --- |
| Insulin-transferrin-selenium-ethanolamine (ITSX) | 1 x | Gibco |
| Penicillin/streptomycin/Glutamine (P/S/G) | 1 x | Gibco |
| HEPES | 10 mM | Gibco |
| Polyvinyl alcohol (PVA) | 1 mg/ml | Sigma |
| Animal free stem-cell factor (SCF) | 10 ng/ml | Peprotech |
| Animal free thrombopoietin (TPO) | 100 ng/ml | Peprotech |
| In Ham’s F-12 Nutrient Mix (Gibco) | | |

**Supplementary File 1b. Lineage cocktail (Biotinylated)**

| **Surface marker** | **Coupled dye** | **Clone** | **Dilution** | **Provider** |
| --- | --- | --- | --- | --- |
| B220 | Biotin | RA3-6B2 | 1:200 | Sony |
| CD3 | Biotin | 17A2 | 1:100 | Sony |
| Gr-1 | Biotin | RB6-8C5 | 1:400 | Sony |
| Nk1.1 | Biotin | PK136 | 1:400 | BD |
| Ter119 | Biotin | TER-119 | 1:400 | Sony |

**Supplementary File 1c. Lineage cocktail (PE-Cy5)**

| **Surface marker** | **Coupled dye** | **Clone** | **Dilution** | **Provider** |
| --- | --- | --- | --- | --- |
| B220 | PE-Cy5 | RA3-6B2 | 1:200 | Sony |
| CD3 | PE-Cy5 | 145-2C11 | 1:400 | Sony |
| Gr-1 | PE-Cy5 | RB6-8C5 | 1:400 | Sony |
| Nk1.1 | PE-Cy5 | PK136 | 1:400 | Sony |
| Ter119 | PE-Cy5 | TER-119 | 1:400 | Sony |

**Supplementary File 1d. Antibody mixture for BM or FL cHSC sorting**

| **Surface marker** | **Coupled dye** | **Clone** | **Dilution** | **Provider** |
| --- | --- | --- | --- | --- |
| Lineage cocktail | As outlined in Table 2. | | | |
| CD48 | FITC | HM48-1 | 1:200 | Sony |
| CD150 | PE-Cy7 | TC15-12F12.2 | 1:200 | Sony |
| CD201 | PE | RCR-16 | 1:200 | Sony |
| Sca1 | PB | E13-161.7 | 1:200 | BioLegend |

* CD41-PerCP-eFluor710 (1:100, Clone eBioMWReg30, eBioscience Cat. #46-0411-82) was included if mentioned in the results.

**Supplementary File 1e. Antibody mixture for cHSC culture analysis and sorting**

| **Surface marker** | **Coupled dye** | **Clone** | **Dilution** | **Provider** |
| --- | --- | --- | --- | --- |
| Lineage cocktail | As outlined in Table 3. | | | |
| CD48 | AF700 | HM48-1 | 1:100 | Sony |
| CD150 | PE-Cy7 | TC15-12F12.2 | 1:200 | Sony |
| CD201 | PE | RCR-16 | 1:200 | Sony |
| cKit | APCeFluor780 | 2B8 | 1:100 | eBioscience |
| Fcer1a | FITC | MAR-1 | 1:200 | eBioscience |
| Sca1 | PB | E13-161.7 | 1:200 | BioLegend |

* In case of analysis of expansion from Fgd5-ZsGreen labeled HSCs, Fcer1a-FITC was excluded.

**Supplementary File 1f. Antibody mixture for PB chimerism analysis**

| **Surface marker** | **Coupled dye** | **Clone** | **Dilution** | **Provider** |
| --- | --- | --- | --- | --- |
| CD3 | AF700 | 17A2 | 1:200 | Sony |
| CD11b | APC | M1/70 | 1:200 | Sony |
| CD19 | PE-Cy7 | 6D5 | 1:400 | Sony |
| CD45.1 | BV650 | A20 | 1:100 | Sony |
| CD45.2 | BV785 | 104 | 1:100 | Sony |
| Gr-1 | PE | RB6-8C5 | 1:400 | Sony |
| Nk1.1 | PB | PK136 | 1:200 | Sony |
| Ter119 | PerCP-Cy5.5 | TER-119 | 1:200 | Sony |

**Supplementary File 1g. Antibody mixture for BM cHSC chimerism analysis**

| **Surface marker** | **Coupled dye** | **Clone** | **Dilution** | **Provider** |
| --- | --- | --- | --- | --- |
| Lineage cocktail | As outlined in Table 3. | | | |
| CD45.1 | BV650 | A20 | 1:100 | Sony |
| CD45.2 | BV785 | 104 | 1:100 | Sony |
| CD48 | AF700 | HM48-1 | 1:100 | Sony |
| CD150 | PE-Cy7 | TC15-12F12.2 | 1:200 | Sony |
| CD201 | PE | RCR-16 | 1:200 | Sony |
| cKit | APCeFluor780 | 2B8 | 1:100 | eBioscience |
| Sca1 | PB | E13-161.7 | 1:200 | BioLegend |

**Supplementary File 1h. Antibody mixture for culture CTV analysis**

| **Surface marker** | **Coupled dye** | **Clone** | **Dilution** | **Provider** |
| --- | --- | --- | --- | --- |
| Lineage cocktail | As outlined in Table 3. | | | |
| CD48 | APC | HM48-1 | 1:200 | Sony |
| CD150 | PE-Cy7 | TC15-12F12.2 | 1:200 | Sony |
| CD201 | PE | RCR-16 | 1:200 | Sony |
| cKit | APCeFluor780 | 2B8 | 1:100 | eBioscience |
| Fcer1a | FITC | MAR-1 | 1:200 | eBioscience |
| Sca1 | BV711 | D7 | 1:200 | Sony |

**Supplementary File 1i. Antibody mixture for BM CTV analysis**

| **Surface marker** | **Coupled dye** | **Clone** | **Dilution** | **Provider** |
| --- | --- | --- | --- | --- |
| Lineage cocktail | As outlined in Table 3. | | | |
| CD45.1 | BV650 | A20 | 1:100 | Sony |
| CD45.2 | BV785 | 104 | 1:100 | Sony |
| CD48 | FITC | HM48-1 | 1:200 | Sony |
| CD135 | PE | A2F10 | 1:100 | Sony |
| CD150 | PE-Cy7 | TC15-12F12.2 | 1:200 | Sony |
| CD201 | APC | eBio1560 | 1:200 | eBioscience |
| Sca1 | BV711 | D7 | 1:200 | Sony |

**Supplementary File 1j. Antibody mixture for Spleen CTV analysis**

| **Surface marker** | **Coupled dye** | **Clone** | **Dilution** | **Provider** |
| --- | --- | --- | --- | --- |
| CD4 | APC-Cy7 | GK1.5 | 1:200 | Sony |
| CD11b | FITC | M1/70 | 1:400 | BD |
| CD19 | PE-Cy7 | 6D5 | 1:400 | Sony |
| CD45.1 | BV650 | A20 | 1:100 | Sony |
| CD45.2 | BV785 | 104 | 1:100 | Sony |
| Ter119 | PE-Cy5 | TER-119 | 1:400 | Sony |

**Supplementary File 1k. List of primers**

| **Primer pair** | **Sequence (5’ – 3’)** |
| --- | --- |
| Pre-culture Forward | TCGTCGGCAGCGTCAGATGTGTATAAGAGACAGGAAGCTGCGCCTGTCATC |
| Pre-culture Reverse | GTCTCGTGGGCTCGGAGATGTGTATAAGAGACAGGTGAACCGCATCGAGCTG |
| Post-culture Forward | TCGTCGGCAGCGTCAGATGTGTATAAGAGACAGTGGAGAACCACCTTGTTGG |
| Post-culture Reverse | GTCTCGTGGGCTCGGAGATGTGTATAAGAGACAGTGCATGGCGGTAATACGGT |
| P5 index N101 | AATGATACGGCGACCACCGAGATCTACAC **TAGATCGC** TCGTCGGCAGCGTC |
| P5 index  S502 | AATGATACGGCGACCACCGAGATCTACAC **CTCTCTAT** TCGTCGGCAGCGTC |
| P5 index S503 | AATGATACGGCGACCACCGAGATCTACAC **TATCCTCT** TCGTCGGCAGCGTC |
| P7 index N701 | CAAGCAGAAGACGGCATACGAGAT **TCGCCTTA** GTCTCGTGGGCTCGG |
| P7 index N901 | CAAGCAGAAGACGGCATACGAGAT **AACGTGAT** GTCTCGTGGGCTCGG |
| P7 index N902 | CAAGCAGAAGACGGCATACGAGAT **AAACATCG** GTCTCGTGGGCTCGG |
| P7 index N903 | CAAGCAGAAGACGGCATACGAGAT **ATGCCTAA** GTCTCGTGGGCTCGG |
| P7 index N904 | CAAGCAGAAGACGGCATACGAGAT **AGTGGTCA** GTCTCGTGGGCTCGG |
